# Salivary Tumour Necrosis Factor-α as a Biomarker in Oral Leukoplakia and Oral Squamous Cell Carcinoma

**DOI:** 10.31557/APJCP.2019.20.7.2087

**Published:** 2019

**Authors:** Deepthi G, S R K Nandan, Pavan G Kulkarni

**Affiliations:** 1 *Department of Oral and Maxillofacial Pathology, Government Dental College and Hospitals, Hyderabad,*; 2 *Department of Oral and Maxillofacial Pathology, Kamineni Institute of Dental Sciences, Narketpally, Nalgonda, Telangana, India.*

**Keywords:** Biomarker- oral cancer- saliva- tumor necrosis factor – α

## Abstract

**Background:**

Oral cancer is one of the life threatening disease which requires an availability of a biomarker for its early detection and also for effective treatment strategies. The current study is done to evaluate the efficacy of one such biomarker i.e. TNF- α as an indicator for oral precancer and oral cancer.

**Objectives:**

To evaluate the efficacy of Tumour necrosis factor - alpha (TNF)-α as a salivary biomarker in histopathologically diagnosed cases of oral leukoplakia and Oral squamous cell carcinoma. To correlate the levels of TNF- α with varying histologic grading in Oral Squamous Cell Carcinoma and dysplasia grading in Oral leukoplakia or Hyperkeratosis.

**Materials and Methods:**

The study group included 90 subjects that were divided into three groups. OSCC (n=30), leukoplakia (n=30) and controls (n=30). Cases were selected based on inclusion and exclusion criteria of the study. Salivary samples were then collected from all three groups. Salivary levels of TNF-α were estimated using Enzyme Linked Immunosorbent Assay (ELISA). The data on concentration gradients obtained were subjected to appropriate statistical analysis.

**Results:**

The results of the present study demonstrated higher levels of salivary TNF-α in individuals with OSCC compared to leukoplakia and healthy control subjects with a high level of statistical significance. ROC curve analysis along with diagnostic parameter calculation also revealed that salivary TNF-α to be a better medium for detecting OSCC. There is also an increase in the salivary TNF-α levels with increase in the histological grade of differentiation in OSCC as well as leukoplakia.

**Conclusion:**

The present study concludes that salivary TNF – α can be used as a prognostic biomarker of OSCC. In view of the elevated levels of TNF – α in saliva of individuals with severe dysplasia, it can also be used to monitor the malignant transformation to leukoplakia to OSCC.

## Introduction

Oral cancers being the most common of Head and neck cancers, accounts for 354,864 new cases and 177,384 cancer related deaths worldwide in 2018. Cancers of lip and oral cavity are highly frequent in southern Asia and also leading cause of death in men of India and Sri Lanka (Bray et al., 2018). Oral cancer comprises about 80–90% of head and neck cancers, with the most common variant being oral squamous cell carcinoma (OSCC) (Osman et al., 2012). The combined influence of genetic and environmental exposure to carcinogens resulting in genetic damage. These accumulated genetic damages contribute to the formation of OSCC which may sometimes predispose from a clinically evident oral potentially malignant disorders (OPMD), the most common being the Oral leukoplakia (Choi et al., 2008). Most of these oral cancers show a strong association with tobacco and smoking habits, which upon chronic exposure results in changes in the oral mucosa resulting in the development of OPMDs and OSCC. 

Despite the development of treatment modalities which can bring about the improvement in the survival and prognosis of oral cancer, it still continues to be the life-threatening disease as there is definite absence of signs which would help in the early detection of OSCC. To date, there are various gold standard management and diagnostic options for oral cancer which are based on biopsy and histopathological analysis. Gold standard procedures are not convenient in screening procedures of oral cancer among high-risk population (Vanja et al., 2015). Hence, it is likely that few biomarkers are needed to predict the evolution of OSCC from an OPMD and also to screen the high-risk patients with tobacco and/or smoking habits. It is of very much importance that the combined early detection and the effective treatment strategies could help in the prevention and the good prognostic outcomes of oral cancer (Juretic et al., 2013).

The development of cytokines as biomarkers for detecting malignancies has helped in realizing the importance of inflammation-mediated carcinogenesis in OPMDs and in oral cancer. The most common of which include tumor necrosis factor alpha (TNF-α), interleukins (IL)-6 and -8, vascular endothelial growth factor (VEGF), IL -4 and -10 (Kaur et al., 2015). TNF – α, a widely expressed pro-inflammatory cytokine, has been shown diagnostic utility in oral cancer and is found more abundant in saliva compared to that in serum (Rhodus et al., 2005).

TNF-α is a pleiotropic, pro- inflammatory cytokine, and is a double-edged sword being the pro and anti- tumorigenic i.e. TNF- α can be cytotoxic to tumor cells, inhibiting the progression of the tumor or causing necrosis and it can also stimulates angiogenesis, proliferation, migration and survival of tumour cells in most cancer cells (Nakano et al., 1999). The constraint in the number of studies conducted to detect the actual response of TNF- α limits the knowledge of its role in oral precancer and oral cancer.

Thus, with the above facts, the objective of the present study was to evaluate the utility and to determine the role of salivary TNF- α in OPMDs and OSCC correlating with the grades of dysplasia and histological differentiation respectively using ELISA technique.

## Materials and Methods

*Study subjects and the source of the data*

The subjects are patients who presented to Kamineni Institute of Dental Sciences, Narketpally, Nalgonda; MNJ Institute of Oncology, Redhills, Hyderabad and Government Dental College, Afzalgunj Hyderabad. The study included 90 subjects that were divided into three groups. Group A included 30 oral leukoplakia patients who were clinically and histopathologically diagnosed into different grades of oral epithelial dysplasia (OED). WHO in year 2005 classified OED as mild, moderate, severe and carcinoma in situ according to the presence and severity of cellular atypia and the architectural features. Group B included 30 patients of OSCC who were clinically and histopathologically diagnosed with varying grades i.e., well / moderately / poorly differentiated oral squamous cell carcinoma. Group C included healthy controls with age, sex match and periodontal status confirmed by Community periodontal index (CPI) matched to that of group A and B. The group A and B individuals were clinically diagnosed and were not currently undergoing or having undergone any form of definitive therapy in the form of surgery, radiation, chemotherapy or any other adjunctive treatments for OSCC. 

**Table 1:** Demographic Data of the Individuals of Three Groups

Data	Controls	Leukoplakia	OSCC
Total no. of cases	30	30	30
Age	20-67yrs	24-73yrs	24- 74yrs
Males	20	26	26
Females	10	4	4
Smokers	-	28	12
Tobacco chewer	-	5	18
Histological differentiation			
Well differentiated	-	-	13
Moderately differentiated			10
Poorly differentiated			7
Histological differentiation			
Mild dysplasia	-	10	-
Moderate dysplasia		10	
Severe dysplasia		10	

**Table 2:** Descriptive Statistics of Individual Groups

Group	n	Mean	Median	Standard deviation	Minimum	Maximum	First quartile	Third quartile
OSCC	30	63.94	43.75	56.05	16.5	253	24.5	77
leukoplakia	30	28.96	21.825	20.94	7.8	91.5	13.8	38
Controls	30	5.75	4.65	3.98	2.15	21.6	3.2	7.2

**Table 3:** Mean Score Analysis of Three Different Groups by Kruskal Wallis Test (Analysis of Variance). *p- value is significant (p<0.01)

Groups	Sample size (n)	Mean	Standard deviation	Sum of ranks	Test statistics (H)	p
OSCC	30	63.94	56.05	2,059.5	61.8754	0.00* (S)
Leukoplakia	30	28.96	20.94	1539		
Controls	30	5.75	3.98	496.5		

**Table 4:** Comparison of TNF – α Levels in Different Grades of Leukoplakia (Oral Epithelial Dysplasia) by One- Way ANOVA Test. ‘p’ value is not significant

	Mild dysplasia	Moderate dysplasia		Severe dysplasia	Total
N	10	10		10	30
∑X	199.8	259.05		399.95	858.8
Mean	19.98	25.905		39.995	28.6267
Standard deviation	12.9976	21.0822		23.5899	20.8798
Source	Sum of squares	Df	F- ratio		p
Between- groups	2114.1132	2	2.71068		0.084565
Within- groups	10528.9305	27			(NS)
Total	12643.0437	29			

**Table 5:** Comparision of TNF – α Levels in Different Grades of OSCC by One- Way ANOVA Test. *p value is significant (p<0.01)

	WDSCC	MDSCC	PDSCC	Total
N	13	10	7	30
∑X	507.2	494.9	906	1908.1
Mean	39.0154	49.49	129.4286	63.6033
Standard deviation	22.2301	25.8839	81.3636	56.282
Source	Sum of squares	Df	f- ratio	p
Between groups	40181.9695	2	10.49642	0.000424*
Within - groups	51680.1602	27		(S)
Total	91862.1297	29		

**Figure 1:**
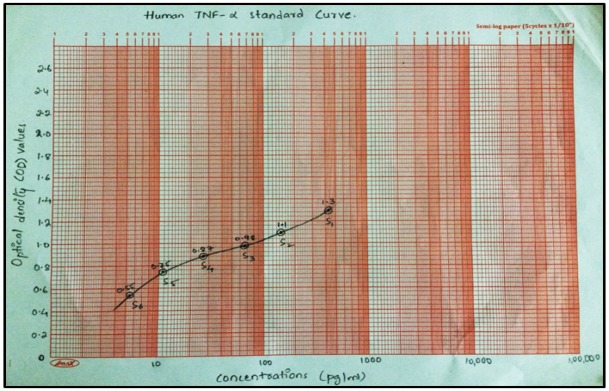
Standard Curve Plotted on a Semi-Log Graph with OD Values on y-axis and Concentrations (pg/ml) of TNF- α on x-axis

**Figure 2:**
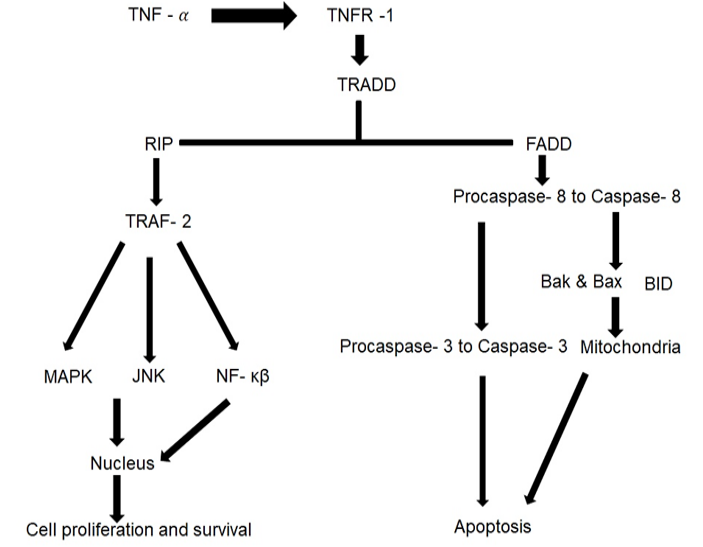
Tumor Necrosis Factor Receptor 1 (TNFR-1) Signalling Pathway

**Graph 1:**
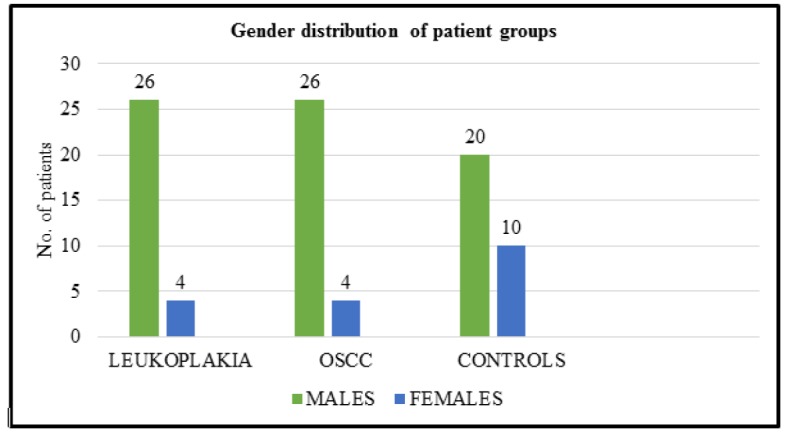
Gender Distribution of Study Groups

**Graph 2:**
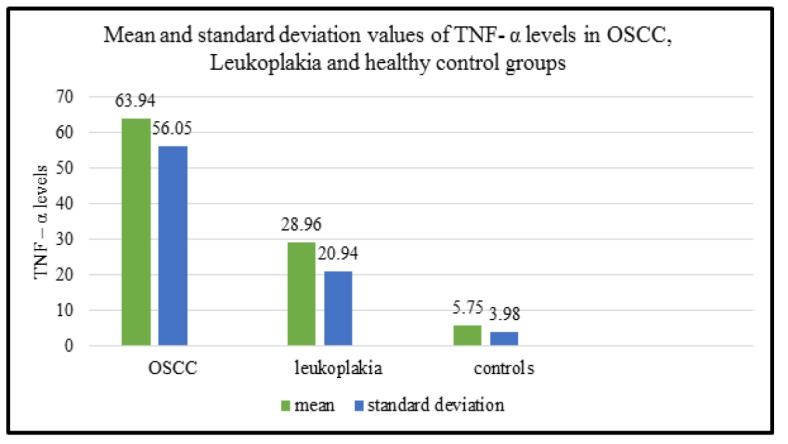
Mean and Standard Deviation Values of TNF- α Levels in OSCC, Leukoplakia and Healthy Control Groups

**Graph 3:**
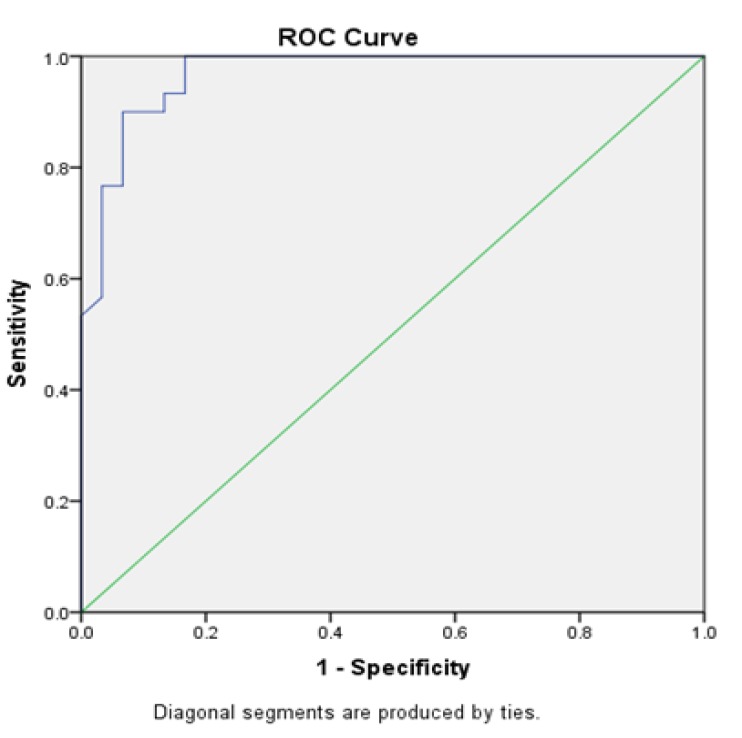
ROC Curve Analysis to Determine the Sensitivity and Specificity of TNF – α Levels in Oral Leukoplakia

**Graph 4:**
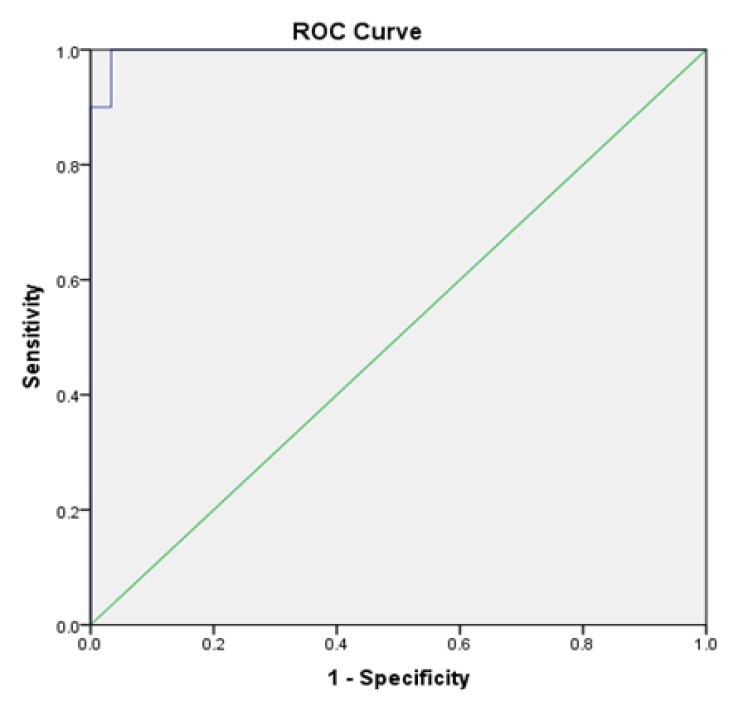
ROC Curve Analysis to Determine the Sensitivity and Specificity of TNF – α Levels in OSCC

*Histopathological grading*

The histopathological specimens were observed under light microscope (4-40 X magnification) by an Oral and Maxillofacial Pathologist expert(s). The histopathological grading was done based on the following grading systems: Leukoplakia- according to WHO grading of Oral Epithelial Dysplasia, OSCC were graded using Broder’s grading system.

The study received Institutional ethical committee approval from Kamineni Institute of Dental Sciences, Hyderabad. Written informed consents were taken from all the participating subjects.

*Exclusion criteria*

Individuals with any other underlying illnesses that would raise levels of cytokines in saliva were excluded. Individuals with history of local therapeutic medications for any other oral lesions or medications such as anti-histaminics, anti-hypertensives, anti-cholinergics, antidepressants, bronchodilators, or pathological dry mouth syndrome, or inability to collect sufficient saliva samples on a reliable basis.

*Saliva collection*

Salivary samples were collected from subjects during 10:00 AM to 12:00 PM under non-stimulatory condition. Participants were guided to refrain from either eating or drinking at least one hour before collection of salivary samples. Subjects were asked to rinse out their mouth with water at least 5 min prior to saliva collection. Samples were obtained by requesting subjects to swallow first, tilt their head forward, and expectorate all saliva into the sterile disposable tubes. The collected saliva is centrifuged at 3,500 rpm for 10 minutes to remove debris. The supernatant is collected into 1.5ml eppendorf tubes. These tubes with the supernatant sample was stored at -80^o^C till further analysis. One freeze thaw cycle was done per sample. 

*TNF- α estimation*

Estimation of salivary TNF- α was done by using DeQuantoTM Human TNF alpha ELISA kit, # QT 4001. The assay was carried out according to the manufacturer’s instructions with some modifications, because the kits were primarily designed for serum samples wherein the concentrations of these constituents are generally high. The technique of ELISA followed here was sandwich method. The method briefly follows: 

The samples/ test specimens were brought to the room temperature. Addition of 100 μl of test/specimen samples to each well of pre - TNF- α antibody coated 96-well microtiter plate, which was s incubated for 1 hour and washed. Followed by addition of 100 μl of detection antibody to each well of the microtiter plate and incubated for 1 hour at ~25°C followed by washing. Then adding of 100 μl of streptavidin - HRP to each well of the microtiter plate and incubate for 30 minutes at ~25°C, followed by adding 100 μl of TMB substrate solution to each well of the microtiter plate and incubated for 15 minutes in dark at ~25°C is done. To stop the reaction, 50 μl of stop solution (2N H_2_SO_4_) to each well was added. The resultant product is yellow. The changes in colour intensity and the absorbance at 450 nm and 600 nm was read using ELISA microplate reader (EMP emperor medical - M201 microplate reader). 

A standard curve was prepared by plotting absorbance readings or optical density (OD) values of standards (S1 S6) against their concentrations (Figure 1). The OD values of the samples of the present study were plotted on the standard curve and the concentrations of the total TNF- α in picograms/millilitre (pg/ml) were calculated in all the three groups.

*Statistical analysis*

All statistical procedures were performed with SPSS version SPSS 19.0 (PASW statistics). Data were tested for normality using Ryan- joiner test. Descriptive statistics like mean, median, interquartile range and standard deviation were calculated. The Kruskal-Wallis analysis of variance and the Mann-Whitney U test were used to evaluate differences between multiple group and unpaired observations respectively.

## Results

*Demographic characteristics*

The present study was conducted to evaluate the role of salivary TNF- α in oral leukoplakia and OSCC using ELISA technique. The age and sex matched 30 control subjects, 30 leukoplakia subjects and 30 OSCC subjects were included. All the oral leukoplakia and OSCC subjects either had tobacco/pan chewing/smoking habits. The oral leukoplakia lesions were from buccal mucosa (n=19), palate (n= 1), tongue (n= 2), floor of the mouth (n= 3) and retromolar trigone (n= 5). Similarly, the OSCC lesions were from buccal mucosa (n=7), alveolar mucosa (n=2), palate (n=4), tongue (n=8), floor of the mouth (n= 4), retromolar trigone (n= 2) and oropharynx (n= 3). 

Based on the histopathological grading of OSCC, 13 (43.33%) patients had well differentiated, 10 (33.33%) had moderately differentiated and 7 (23.33%) had poorly differentiated lesions. Similarly, based on the histopathological grading of the leukoplakia (oral epithelial dysplasia), 10 (33.33%) patients had mild epithelial dysplasia, 10 (33.33%) patients had moderate epithelial dysplasia and 10 (33.33%) patients had severe epithelial dysplasia. The study population in all the groups were predominantly males and the gender wise distribution of different groups is illustrated in [Graph 1]. Demographic data of the individuals of three groups are summarized in (Table 1). 

*Comparison of concentration of salivary TNF - α levels among different groups*

The mean and standard deviation of the salivary levels of TNF-α in OSCC, leukoplakia and control groups are calculated and are 63.94 ± 56.05, 28.96 ± 20.94 and 5.75 ±3.98 respectively. The comparison of these values among the three groups are illustrated in (Graph 2). The descriptive statistics of levels of salivary TNF-α in three groups with OSCC group showing - median: 43.75; range: 16.5- 253, leukoplakia - median: 21.825; range: 7.8 – 91.5 and control group - median: 4.65; range: 2.15 – 21.6 (Table 2).

The test for mean score analysis (KruskalWallis analysis of variance) procedure is used to compare mean scores of more than two groups. The test showed a highly significant (p = 0.00) difference between the two groups (p<0.01) (Table 3).

*Comparison of TNF – α levels in different grades of OED (Leukoplakia)*

Among 30 patients of leukoplakia, 10 (33.33%) patients had mild epithelial dysplasia, 10 (33.33%) patients had moderate epithelial dysplasia and 10 (33.33%) patients had severe epithelial dysplasia. Comparison of salivary TNF – α levels in these different grades of oral epithelial dysplasia (leukoplakia) is done by using one – way ANOVA test, the results showed that there is no significant difference among different grades dysplasia. The p value of 0.084565 which is non- significant (p>0.01) is obtained (Table 4). 

*Comparison of TNF – α levels in different grades of OSCC*

For 30 patients in OSCC, 13 (43.33%) patients had well differentiated, 10 (33.33%) had moderately differentiated and 7 (23.33%) had poorly differentiated lesion. Comparison of salivary TNF – α levels in these grades of OSCC is done by using one – way ANOVA test, which showed a highly significant increase in PDSCC compared to MDSCC and WDSCC with a p value of 0.000424 (p<0.01) (Table 5). 

*ROC curve analysis*

ROC curve analyses revealed that salivary TNF-α could serve as valuable biomarker for differentiating leukoplakia (oral epithelial dysplasia) from healthy controls with an area under the curve (AUC) of 0.968 (95% Confidence Interval: 0.930 – 1.000). Significant difference between salivary TNF - α levels of leukoplakia and controls with AUC was found to be 0.00 (p<0.001). At the cut- off value of 10.25 pg/ml, the salivary TNF-α showed a sensitivity and specificity of 90 % and 93.3% respectively (Graph 3). 

ROC curve analyses revealed that salivary TNF-α could serve as valuable biomarker for differentiating OSCC from healthy controls with an area under the curve (AUC) of 0.997 (95% Confidence Interval: 0.989 – 1.000). Significant difference between salivary TNF - α levels of OSCC and controls with AUC was found to be 0.00 (p<0.001). At the cut- off value of 15.100 pg/ml, the salivary TNF-α showed a sensitivity and specificity of 100 % and 96.7% respectively (Graph 4).

*Diagnostic utility*

The results of the present study demonstrated higher level of salivary TNF-α production in OSCC while comparing to premalignant disorder and healthy control subjectswith a high level of statistical significance. The comparison of mean levels between different grades of oral epithelial dysplasia showed non- significant increase from mild to severe dysplasia. The mean values between different grades of OSCC showed a highly significant increase in salivary TNF-α levels from WDSCC to PDSCC. ROC curve analysis along with diagnostic parameter calculation reveals salivary TNF-α to be a better medium for detecting OSCC.

## Discussion

OSCC comprises of the tumour epithelium and the surrounding connective tissue stroma which constitutes the tumour microenvironment (TME) within which varying populations of mesenchymal cells, extracellular matrix, and inflammatory cells are present (Koontongkaew, 2013). TME provides the cross- talk between the tumour cells and the stromal elements like inflammatory cells & cancer associated fibroblasts contributing to the development, growth, invasion and metastasis of the tumour. This crosstalk is mediated by the generation of several inflammatory cytokines such as TNF – α. These cytokines attract and recruit more inflammatory cells to the tumour microenvironment to further enhance the proliferation and survival of genetically altered tumour cells (Curry et al., 2014).

TNF-α receptors are expressed on both epithelial and stromal cells. TNF receptor 1 (TNFR-1) and TNFR-2, TNFR-1 is ubiquitously expressed while TNFR-2 is expressed in immune cells. TNF binds to TNF receptor I and/or II (TNFR1 and 2) to activate a receptor signalling complex, which consists of several death-domain-containing proteins, and to transduce two major intracellular signalling pathways: Nuclear factor kappa-light-chain-enhancer of activated B cells (NF-kB) and the caspase cascade (van Horssen et al., 2006). Ligand binding to TNFR1 induces activation of the activator protein -1 (AP1) transcription factors or IκB kinases (IKKs) that, in turn, activate NF-κB. NF-κB activation also importantly induces negative regulators of apoptosis such as FLIPL, BCL-2 and superoxide dismutase. If NF-κβ activation is inadequate, apoptosis is mediated through caspase 8 and and mitochondrial pathways (Balkwill et al., 2009) (**Figure 2**).

In the present study, the mean values of concentrations of salivary TNF- α are greater in OSCC (63.94 ± 56.05) compared to that of OED (28.96 ± 20.94) and control groups (5.75 ±3.98). These values are statistically significant. These results were in concordance with the studies of Rhodus et al., (2005); Saheb Jamee et al., (2008); Juretic et al., (2012); Krishnan et al., (2014).

Rhodus et al., (2005) showed that NF-κB-dependent cytokines i.e TNF-α, IL-1α, IL-6, and IL-8 were elevated in the whole unstimulated saliva of subjects with OSCC compared with PML and controls. ie. TNF- α levels of OSCC are 28.9 ± 14.6, PML with 10.5 ± 7.4 and controls with 3.0 ± 1.9. They have concluded that it is possible that the overexpression of NF-κB correlates with the over-expression of these pro- inflammatory, pro- angiogenic cytokines which may result in the progression of oral cancer from OPMD (Rhodus et al., 2005). SahebJamee et al., (2008) studied the concentrations of salivary TNF-α, IL-1, 6 and IL- 8 using ELISA and concluded that the concentration of salivary cytokine in OSCC was higher than control group with mean values of OSCC with 35.2 ± 51.8 pg/ ml and controls with 4.1 ± 2.1 pg/ml. 

Juretic et al., (2012) conducted a study to determine the salivary concentrations of TNF-α in patients with premalignant lesions (PML) and malignant lesions using ELISA and observed that the mean values of TNF-α are greater in OSCC (0.739 ± 0.176) than PML (0.601 ± 0.178) and controls (0.013 ± 0.033) (Juretic et al., 2012). Krishnan et al., (2014) conducted a study to validate the efficacy of TNF- α in diagnosing premalignant oral lesions and OSCC. The salivary TNF- α levels are greater in OSCC with a mean value of 311.9+ 95.3 than PML with mean value of 136.8+59.6 and controls with 4.5+ 2.5. 

There is also a statistically significant increase in the mean values of salivary TNF – α levels in different grades of OSCC is observed in the present study. The median values of salivary TNF – α levels of WDSCC, MDSCC and PDSCC are as 32.5, 41.75 and 150 pg/ml respectively. These results are in concordance with Krishnan et al., (2014); where evaluation of correlation of the levels of salivary and serum TNF- α with clinic-pathologic factors is done.

Though there are various studies correlating the concentration of the TNF- α levels with histological differentiation of OSCC, but there are very limited studies correlating with the degree of dysplasia. So, the present study addresses that issue. There is an increase in the mean values of salivary TNF – α levels in different grades of OED from mild dysplasia to severe dysplasia, but the values are not statistically significant. 

This altered cytokine responsiveness is tightly associated with the development of oral cancer. In normal cells, stimulation with proinflammatory cytokines leads to growth inhibition, while in oral cancer cells stimulation with proinflammatory cytokines leads to up regulation of posi¬tive cell cycle regulators such as nuclear factor kappa B (NF-κB). Various studies have confirmed that aberrant activation of NF-κβ has been implicated in the development and progression of human cancers, including head and neck squamous cell carcinoma. This results in upregulation of proinflammatory cytokines, proangiogenic factors and anti- apoptotic factors responsible for the tumour growth. Likewise, the constitute production of TNF- α, will be associated with tumour prognosis (Chen et al., 1999).

The sensitivity and specificity values of TNF – α in OSCC are 100 % and 96.7% respectively and sensitivity and specificity values of TNF – α in OED are 90 % and 93.3% respectively. In reference to these above mentioned values the validity and reliability are very high indicating that salivary TNF – α can be used as a biomarker in OSCC and OED. Though salivary markers could not identify the location from which the tumor originated, but they can identify the individuals at risk. These kind of salivary tests can be managed by non- specialists in remote areas as a screening tool to refer the patients affected for careful evaluation to detect the early stage diseases. Saliva tests can also be used to monitor patients in postoperative management of OSCC and may prove beneficial in chemoprevention trials. But testing for biomarkers in body fluids must be done in a prospective, blinded fashion in clinical settings requiring definite detection of disease. The efficiency of these biomarkers can only be estimated only based on a well-designed, prospective multi-institutional trial with larger sample size.

As the present study found that there is an increase in expression of salivary TNF- α levels from controls to leukoplakia to OSCC, this reinforces the hypothesis of TNF- α being a pro- tumorigenic. The present study also supports the utility of saliva in biomarker estimation and in evaluating TNF- α as a prognostic marker, also as an indicator for the neoplastic transformation from OPMD to cancer and in mass screening programmes. 
